# Export dynamics as an optimal growth problem in the network of global economy

**DOI:** 10.1038/srep31461

**Published:** 2016-08-17

**Authors:** Michele Caraglio, Fulvio Baldovin, Attilio L. Stella

**Affiliations:** 1 Dipartimento di Fisica e Astronomia and sezione INFN, Università di Padova, Via Marzolo 8, I-35131 Padova, Italy

## Abstract

We analyze export data aggregated at world global level of 219 classes of products over a period of 39 years. Our main goal is to set up a dynamical model to identify and quantify plausible mechanisms by which the evolutions of the various exports affect each other. This is pursued through a stochastic differential description, partly inspired by approaches used in population dynamics or directed polymers in random media. We outline a complex network of transfer rates which describes how resources are shifted between different product classes, and determines how casual favorable conditions for one export can spread to the other ones. A calibration procedure allows to fit four free model-parameters such that the dynamical evolution becomes consistent with the average growth, the fluctuations, and the ranking of the export values observed in real data. Growth crucially depends on the balance between maintaining and shifting resources to different exports, like in an explore-exploit problem. Remarkably, the calibrated parameters warrant a close-to-maximum growth rate under the transient conditions realized in the period covered by data, implying an optimal self organization of the global export. According to the model, major structural changes in the global economy take tens of years.

Issues concerning growth are of central importance in economic theories. Standard tools for analyzing economies evolution are empirical regressions, which allow to extract trends, estimate fluctuations and produce forecasts. When data are referring to a multitude of different productions, investments, or countries, one faces the problem of understanding how the relations among a large number of entities influence the overall dynamics. The problem then does not reduce to the estimation of individual or average trends, but also involves the identification and quantification of collective effects possibly influencing such trends and fluctuations. Through the estimation of these effects we learn something about the organization and functioning of the economy. In particular, we can establish by which mechanisms and up to what extent the collective effects contribute to the overall growth.

Such goals are pursued here by the construction of a minimal model describing how the values of various global exports evolve in time. Theoretical modeling based on stochastic differential equations is widely applied since long to different problems with growth-related features, like population and evolutionary dynamics^1^, portfolio strategies^2^, interface growth^3^ or optimal pinning of vortices by random defects in materials^4^. In economic settings, when for example representing the dynamics of activities in various sectors, *i*, one of the aspects to be modeled concerns the alternance of favorable and unfavorable conditions for growth of the sector’s aggregated value *Z*_*i*_, due to raw material prices, innovations, etc. This can be done by introducing a multiplicative random noise term, *η*_*i*_ *Z*_*i*_, in the expression of the time derivative of *Z*_*i*_, with *η*_*i*_ representing a Gaussian noise with zero average. Since economical trends are characterized by a typical duration *τ*, the noise terms must preserve some memory of the past values and thus be correlated in time. In addition, the representation of an intertwined economy demands for the existence of proper links connecting different sectors. Hence, the dynamical modeling must also include coupling parameters *J*_*ij*_ describing the shift of resources from production *j* to production *i*. Following ref. 5, one then may write.


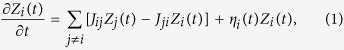


with


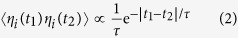


(in the limit *τ* → 0, Brownian noise, *δ*-correlated in time, is recovered). Interestingly, the combination of noise *η*_*i*_ correlated in time and coupling terms *J*_*ij*_ may induce a nonzero average growth even in the absence of deterministic trends driving the single productions^5^. Thus, a most relevant question arises of identifying the optimal coupling conditions which maximize the average growth for a given noise level. These conditions should realize the best compromise between maintaining investments in the same sector or shifting them to other ones, providing an instance of the exploration-exploitation tradeoff problem^5^,^6^,^7^. Previous studies of this problem have been restricted to simplified situations, where the *Z*_*i*_ play equivalent roles and the noise terms, albeit correlated in time, are not cross-influencing each other. Moreover, the conditions of the explore-exploit dilemma have been discussed for asymptotically long times only^5^. Our ambition here is to set up a sensible model adequate to address the complexity of real economic data and to investigate how the exploration-exploitation issue is possibly posed at the global economy level and at accessible, finite times.

Network structures are well established tools^8^ to address economic complexity, especially for growth-related issues^9^,^10^,^11^,^12^,^13^,^14^. Among the goals of the network approach is that of identifying by data-analysis factors influencing the growth potential of a given country, or that of explaining why, in spite of globalization, certain countries are not able to develop productions which would substantially increase their Gross Domestic Product. Within these contexts, exchanges among countries can be represented by a network (World Trade Network) in which countries themselves are the nodes, and links are weighted by the existing amount of trade^9^,^15^. Another possibility is that of considering the exchanged products as nodes, while the strength of the links represents, e.g., the proximity of pairs of products they connect–a measure of the capability of a generic country to produce them simultaneously at a significant level^11^. Based on such networks various schemes for assessing the fitness and the growth prospects of different countries have been recently proposed^11^,^12^,^13^,^14^. However, dynamical considerations developed within these approaches do not rely on the formulation of an autonomous dynamical model with explicit time dependence, as proposed here.

A key feature revealed by our empirical analysis is that, apart from few exceptions, on average the set of exported products displays in time a stable ranking in terms of monetary values. This circumstance enables us to identify a nontrivial network structure which appropriately rules the dynamics. Based on our model we are able to infer from the data basic features of the observed exports dynamics, which include the ingredients of an explore-exploit problem. Besides quantifying the contribution to the growth determined by the coupling between products, we characterize, in an average sense, the time-scale and the extent to which favorable and unfavorable conditions influence each production. Useful insights are also provided about the global response to changes of economic fundamentals redefining the exchange network structure.

## Export-value dynamics

We use international trade data furnished by the National Bureau of Economic Research^16^ to extract yearly exports of 219 product classes on the basis of the Standardized International Trade Code at 3-digit level (SITC-3), in a period of 39 years from 1962 to 2000. This database is corrected for discrepancies among records provided by the exporting and importing countries. The exports are reported in terms of their value in US-dollars. In this respect, one of the factors to be taken into account in estimating growth is the contribution due to the rate of inflation/deflation of the dollar in each year (see below).

**Figure 1:**
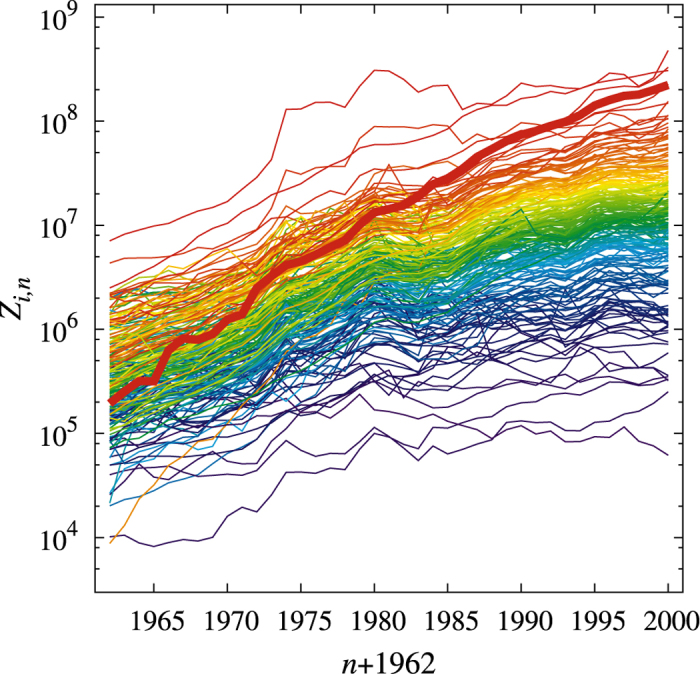
Time evolution of the yearly global value of exported products from 1962 to 2000. Electronics goods are highlighted in thicker line.

We consider for each product class the aggregate export realized by all countries in one year. Let us call *Z*_*i*,*n*_ the total value of product *i (i* = 1, 2, …, 219) exported in the year *n (n* = 0, 1, 2, …, 38). Figure 1 reports these quantities as a function of *n*. The entries for each *i* are reported with a color whose wavelength is proportional to the fraction *z*_*i*_ of the value of product *i* over the global yearly export, averaged on the last 10 years covered by the database:


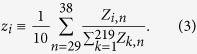


It is evident in Fig. 1 that the various lines, while indicating an approximate average exponential growth, up to fluctuations and a few exceptions do not cross and mix much over the whole period: a rainbow image is perceived. One of the exceptions is represented, e.g., by electronic products, which in the first part of the examined period display a rapid and substantial raise of value, which stabilizes then at a sensibly higher rank than the initial one. Such an effect reflects the great technological advances after the sixties and seventies. Similar transient behaviors occur for other products. This motivates why in Eq. (3) we focused on the last 10 years of the database, as better representative of a stabilizing ranking.

Switching to continuous time *t* in year units (with *t* = 0 corresponding to 1962) as a convenient choice for building up our model, we now indicate by *Z*_*i*_(*t*) the export-value of product *i* in the year preceding *t. Z*_*i*,*n*_ gives a discrete representation of this function in 39 points. The choice of continuous time is legitimated by the fact that yearly records are the result of changes at much shorter time scales. Conceivably, in the simplest model one could think about, *Z*_*i*_(*t*) would perform a sort of geometric Brownian motion, exponentially increasing at a rate *μ* (approximately common to all *i*) and with fluctuations of amplitude *σ* (volatility). This would be consistent with the fact that Fig. 1 displays, for different goods *i*, akin fluctuations and a similar, almost constant average logarithmic slope. However, trajectories of the various products would not tend to the ranking shown by real data, and within such a model the rainbow effect would be progressively lost rather than being progressively emphasized. As anticipated in the Introduction, mechanisms through which different exports influence each other can be specified by a matrix **J** = (*J*_*ij*_)_1≤*i*,*j*≤219_ describing the transfer of value from export *j* to export *i*. So, we generalize Eq. (1) to


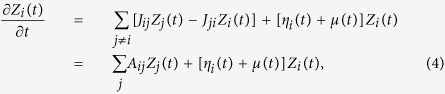


where the rate matrix **A**, with elements *A*_*ij*_ = *J*_*ij*_ for *j* ≠  *i* and *A*_*ii*_ ≡ −∑_*j*≠*i*_*J*_*ji*_, is analogous to the transition matrix defined for continuous-time Markov chains^17^. Here *μ*(*t*) represents a deterministic drift, accounting for the average growth of the exports (including the inflationary one) in the absence of mutual influences. *η*_*i*_(*t*) is a multiplicative noise source representing the variability of conditions faced by different products at different times. Eq. (4) is invariant with respect to changes of the monetary unit for the *Z*_*i*_-values^18^.

For the purposes of the present work, in Eq. (4) **J** does not exclusively represent direct shifts of investments between different sectors of export. More generally, **J** also reflects transfers of resources which are necessary for the production or the utilization of goods. For instance, the increase in the export of cars is likely to determine an increase in the export of oil, even if the two products do not represent a typical alternative for investors and/or producers. As a consequence, the character of the explore-exploit problem based on **J** does not entirely depend on strategic investment policies^6^,^7^, but also reflects intrinsic allocation constraints determined by the economy structure. In most applications (see, e.g.^5^, and references therein), the interaction matrix **J** is short-range and reflects the regularity of a lattice, so that the various *Z*_*i*_’s turn out to be equivalent. In our case different goods are evidently not equivalent, so **J** must play the important role of establishing and maintaining the observed ranking of the various *Z*_*i*_’s. Indeed, in the absence of noise terms the solutions of Eq. (4) tend to a long-time attractor in which the ranking of the *Z*_*i*_’s is determined by the kernel of **A** itself^19^. We can exploit this feature by choosing


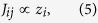


since in this way *z*_*i*_ itself would be in the kernel. With the last choice, it is easy to see that on average the ranking approached by the solutions of Eq. (4) is the one imposed by *z*_*i*_ itself and thus manifested by data. Equation. (5) has also the appeal of being consistent with the gravity law, often used for estimating transfer rates in economics^20^,^21^: it is plausible for the shift of resources from export *j* to export *i* to be proportional to *Z*_*i*_(*t*)*Z*_*j*_(*t*), with *Z*_*i*_(*t*) and *Z*_*j*_(*t*) acting like masses in a gravity-force law. The specific choice in Eq. (5) accounts for such a proportionality in a time-averaged sense. The gravity law has an additional ingredient, an inverse proportionality to a power law of the “distance” between the products. In economic applications, distance is often an elusive concept^22^. In our case, it should measure how far is a given product from another one to the purpose of directly affecting its export. One possibility is that of focusing on the empirical correlation existing between the simultaneous variations of the exports of two products as a proxy to their inverse distance. Thus, we replace the notion of inverse-distance with the modulus |*c*_*ij*_| of the empirical correlator of the logarithmic returns *R*_*i*,*n*_ = ln(*Z*_*i*,*n*_/*Z*_*i*,*n*−1_):


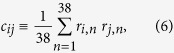


where 
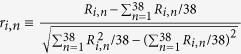
 is the normalized return. Notice that since Eq. (6) involves the product of empirical returns at the same year *n, c*_*ij*_ carries no information about time-correlations. It only encodes the cross-correlations of the products exports at the same time, averaged over the whole period covered by the dataset. In summary, we thus write


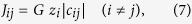


where *G* is a coupling constant, and the factor |*c*_*ij*_| takes into account how much the variations of product *j* influence those of product *i*. The coupling matrix **J** defines a weighted, oriented network connecting as nodes the various products. The plausibility of the form adopted here in Eq. (7) has to be ultimately tested by comparison with the data.

In Eq. (4) *η*_*i*_ is a zero-average Gaussian noise term, describing growth or decay conjunctures for the product-value *Z*_*i*_. These random occurrences are expected to be correlated both in time (in order to represent the existence of opportunity or crisis trends) and among products (since these trends often affect simultaneously more than one export):


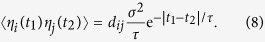


The parameter *τ* is thus defining the typical opportunity or crisis duration, while *σ* weights the importance of the stochastic part of the dynamics. In most growth models^1^,^4^,^5^,^23^ it is generally assumed *d*_*ij*_ = *δ*_*ij*_. We verified however that such an assumption prevents Eq. (4) from quantitatively reproducing the correlator structure *c*_*ij*_ detected empirically. Instead, by choosing *d*_*ij*_ = *c*_*ij*_, the main features of the empirical correlators are recovered to a satisfactory extent when simulating the calibrated model.

The term *μ*(*t*) in Eq. (4) is the deterministic component of the growth rate, valid for each export independently of the existence of the transfer network. In principle, one should consider deterministic trends to be both product-specific and time-dependent. Moreover, they should include an inflationary component beside the real one. In favor of parameter parsimony, here we regard instead *μ*(*t*) as an average growth rate 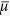 common to all products of the global economy, and we further restrict its time-dependence to the inflationary component *I*(*t*) alone:


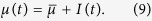


Since *I*(*t*) can be read as an yearly step-wise function from the Consumer Price Index reports^24^ (see Table 1), in such a way we are thus left with the free parameter 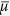, describing the network-independent average global growth rate over the observed time-span. One of our goals is its estimation. This is not trivial, as part of the empirical growth may stem from the capability of the network to spread opportunity trends among different exports.

## Results and Discussion

As explained in the Methods section, a calibration procedure applied over the dataset allows us to determine the four parameters *G, σ, τ*, 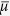, in ordered sequence. Our first result is the reproduction of the basic qualitative features displayed in Fig. 1. Taking into account that some characteristics of the dataset are embedded in the model definition (namely, the last 10-year ranking in *z*_*i*_ and the cross-correlation structure in *c*_*ij*_), this result provides primarily a consistency test. However, the model itself offers a clue for the complexity of the data through four parameters and as such it conveys novel information. In particular, we will show below the existence of an optimal value *G* for maximum growth. Moreover, since the time correlation structure is not *a priori* contained within the model, non-trivial insights about the response properties follows.

**Figure 2:**
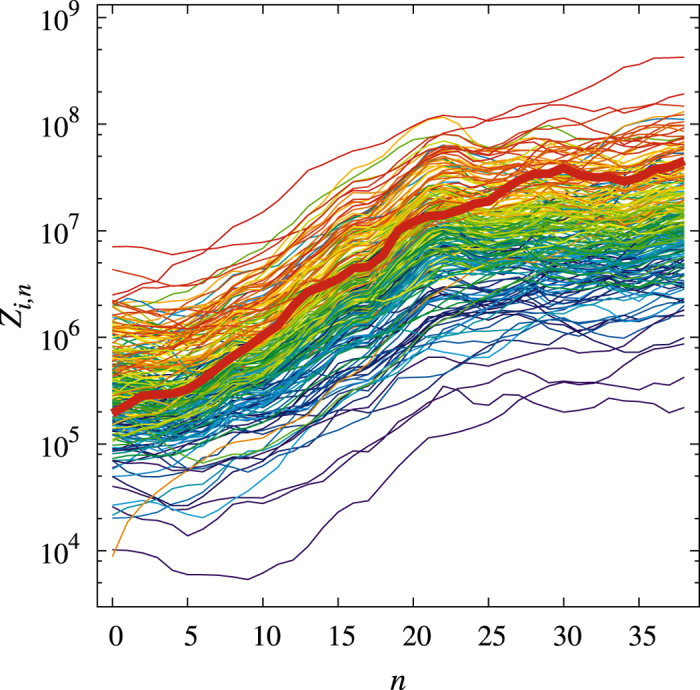
Model time-evolution of the export values starting from the real 1962 initial conditions. Color wavelength is assigned as in Fig. 1. Electronics goods are highlighted with a thicker line.

**Figure 3:**
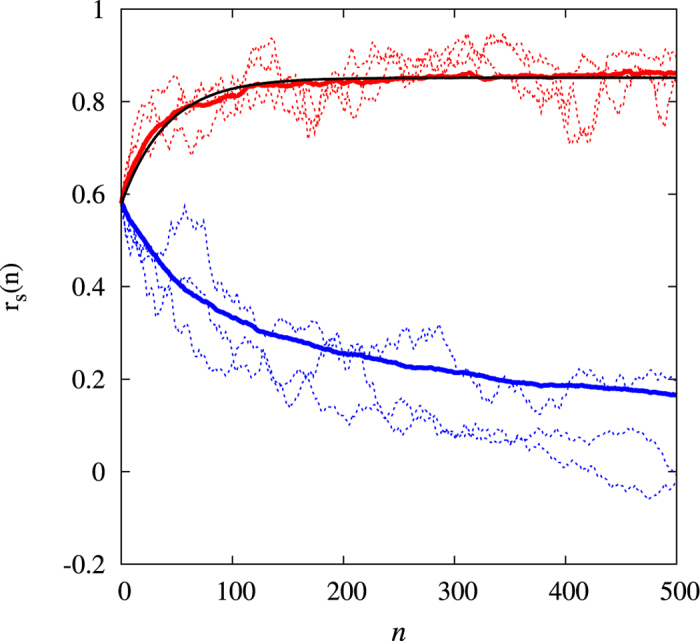
Time evolution of the Spearman’s rank correlation coefficient. The initial condition *Z*_*i*_(0) = *Z*_*i*,0_ corresponds to the situation in 1962. Full red line: average over 100 dynamical evolutions with calibrated parameters. Dotted lines refer to single dynamical realizations. The full black line is a best exponential fit based on Eq. (10) and yielding *τ*_*s*_ = 41 y. The parameter *G* is set to zero for the blue lines.

Not only the “rainbow” effect is replicated by the calibrated-model time evolution reported in Fig. 2, but even specific exports (like electronic products) which in 1962 were out of ranking, in the last years of the evolution are attracted to a proper position. A quantitative way of assessing such a convergence is to consider the Spearman’s rank correlation coefficient −1 ≤ *r*_*s*_ ≤ 1^25^, between results of the model time evolution and the empirical *z*_*i*_’s in Eq. (3). When *r*_*s*_ = 1, the two compared rankings coincide; if *r*_*s*_ = −1, their ordering is opposite. Starting with the 1962’s initial data, *Z*_*i*_(0) = *Z*_*i*,0_, Fig. 3 displays the time evolution of the Spearman’s rank correlator *r*_*s*_(*t*). While for *G* ≠ 0 it is evident the role played by *J*_*ij*_ in attracting the initial data to the proper ranking, the fact that *r*_*s*_(∞) remains smaller than one is due to the random fluctuations. This ranking is also consistent with a very common stylized feature in Economy. Indeed, data analysis applied to both the model and the empirical records reveal that the export value is distributed among the various *Z*_*i*_’s according to a Pareto-type power-law^18^.

The analysis of the Spearman coefficient is also revealing in terms of the time-response to changes in the economy fundamentals which may sensibly alter the product ranking (changes of the transfer rates *J*_*ij*_ in our model). Indeed, the average behavior of *r*_*s*_(*t*) is well approximated by the exponential law


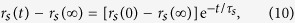


with a characteristic time, within our calibrated model, of 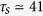 y (see Fig. 3). One could equivalently say that structural changes in the global economy implying a replacement of the dominant products (like, e.g., those required to transform an oil-based economy into a green economy) need tens of years to become effective. In general, dynamical evolutions of our model starting from the 1962 historical initial conditions show that *τ*_*s*_ is inversely proportional to the transfer-rate parameter *G*, at fixed values of the other parameters.

**Figure 4:**
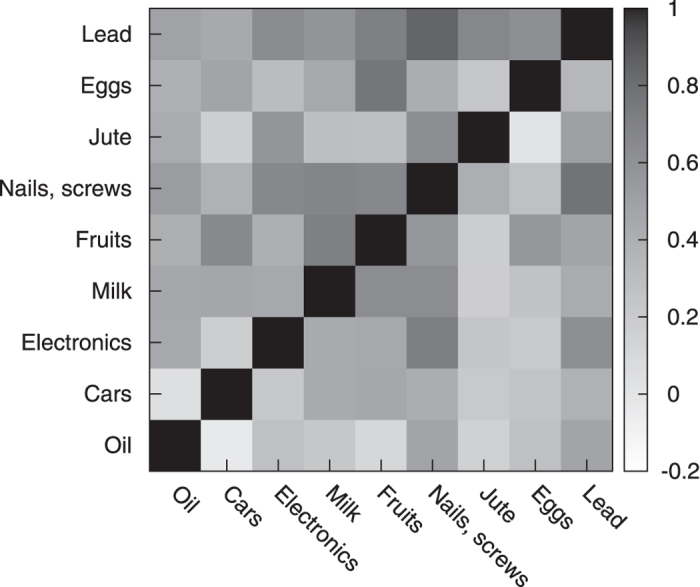
Comparison between historical correlators (above diagonal) and correlators obtained by averaging over twenty histories generated from the calibrated model (below diagonal). The correlators pertain to 9 exports equally divided among low, medium and high ranking. A perfect agreement would imply a diagonal-symmetric plot.

Another result supporting the consistency of the model concerns the cross-correlators *c*_*ij*_ that one can reconstruct from the numerical simulations. In Fig. 4 we compare the correlators estimated from the historical data of a subset of nine exports (boxes below the diagonal) with those obtained by numerical simulations of the calibrated model with historical initial conditions (boxes above the diagonal). On purpose, three of the exports are chosen in the low, three in the medium, and three in the high rank region. Notice the high symmetry of the plot with respect to the diagonal.

**Figure 5:**
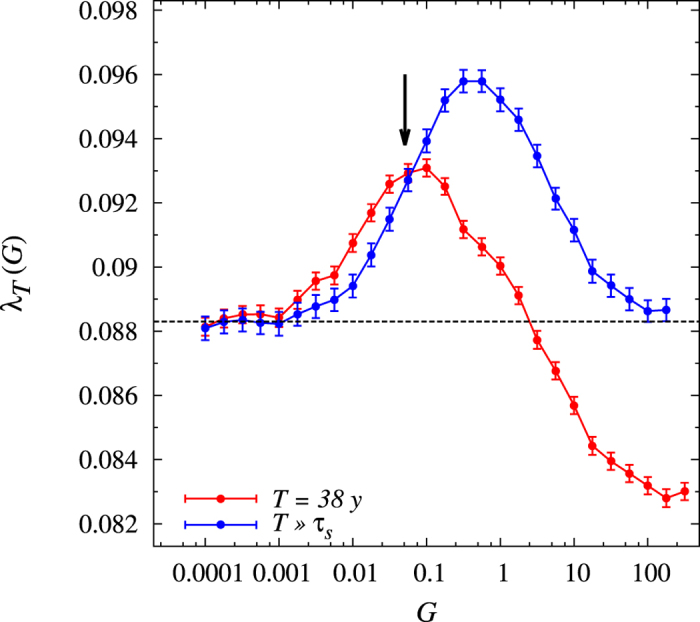
Average growth rate *λ*_*T*_ as a function of *G*. The red line refers to *T* = 38 y, the blue line to the asymptotic growth rate obtained with 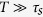. The arrow indicates the calibrated value *G* = 0.051 ± 0.005 y^−1^. The dashed line corresponds to the average deterministic growth 
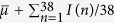
.

An intriguing feature of the model in Eq. (4) is that not all of the observed growth comes from the deterministic rate *μ*(*t*). Indeed, in view of the multiplicative nature of the terms in the equations, local favorable stochastic fluctuations should spread out the network more efficiently than negative random trends, enhancing the average growth. This complies to the dilemma between exploiting a local product’s opportunity (of average intensity *σ* and expected duration *τ*) and exploring other products’ chances throughout the network by transferring part of the local value (at a rate controlled by *G*)^5^. Given the calibrated value for *σ, τ*, and 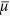, we studied the average growth rate over the time *T*,


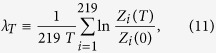


as a function of the transfer coupling constant *G*. If 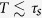, Eq. (11) defines what we call a transient growth rate; it is only in the limit 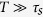 that *λ*_*T*_ becomes the asymptotic growth rate associated to a steady-state evolution. In Fig. 5 we report the curves corresponding to *λ*_*T*_, with *T* = 38 y (red) and 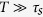 (blue). As *G* increases, *λ*_*T*_ increases with respect to the average deterministic growth rate and, after reaching a maximum, decreases. For large values of *G* the transient growth rate becomes even smaller that the deterministic one. This effect, which does not show up for the steady-state growth rate, is due to specificities of the initial conditions which imply some large transfers in the initial stages of the dynamics. Remarkably, the transient growth-rate attains its maximum very close to the value of *G* ≃ 0.05 y^−1^ obtained in our calibration. Taking into account the weight of the links, ∑_*i*,*j*≠*i*_*J*_*ij*_/*N*, this corresponds to an average yearly transfer of 2% in value for each product. We thus conclude that according to our model the global economy network appears to be self-organized to guarantee close-to-maximum transient growth conditions. On the other hand, on much longer times (corresponding to the stationary regime) *G* should be one order of magnitude larger than the calibrated one for optimal growth conditions. Of course, the features of this stationary regime depend on the assumption of the absence of major changes in the structure of the economy; indeed, the latter could modify the link-weights *J*_*ij*_.

**Figure 6:**
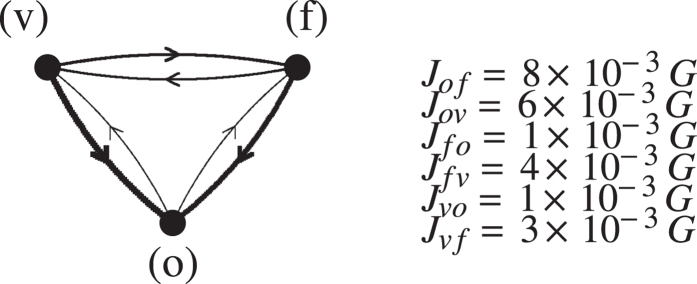
Weighted links among vegetables (v), fruits (f), oil (o) goods according to Eq. (7).

The above results descend from the structure of our model, which explicitly singles out a deterministic component of the growth. In addition, they crucially depend on the peculiar structure of our network of transfer rates primarily dictated by the rank order. It is worth illustrating with an example the specificity of this network. In Fig. 6 we plot the weighted, oriented links among vegetables, fruits, and oil, according to Eq. (7), with heavier lines indicating larger transfer rates. Contrary to naive expectation, the links of both vegetables and fruits to oil are much stronger than those between vegetables and fruits themselves. This emphasizes the need of value transferring from agriculture goods to oil, involved for instance in a petrol-based agriculture production.

## Conclusions

We proposed an autonomous dynamical model describing the evolution of the value of product exports within a global-economy complex network. Minimality has been an obliged feature in order to highlight cooperative endogenous mechanisms underlying economic growth. Specifically, in our equations we singled out a deterministic growth term including inflationary contributions, a stochastic one representing the alternance of favorable and unfavorable conditions to the development of each export class, and finally a network of value-transfers among products. This last ingredient has been successfully identified primarily through a ranking-based criterion. The stochastic component is characterized both by time-correlations associated to the duration of economic trends and by product cross-correlations reflecting, e.g., the existence of economy sectors in which several exports are simultaneously involved. In spite of its parsimonious character, the model provides realistic estimates of fluctuation properties, characteristic response times, and average growth rates. It also provides an evaluation of the average percentage of value transfer for the exports. Importantly, through the distinction between 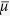 and the empirical growth rate, it characterizes which part of the growth can be ascribed to transfer mechanisms determined by investments and structural interdependences.

We could verify that the global growth complies with the typical conditions of an explore-exploit problem. The optimal solution for such a problem depends on both structural interdependences among products and strategic investment choices. Noteworthy, the calibrated network couplings realize close-to-optimal conditions for maximal average growth in the period covered by data. Thus, for the prevailing correlated noise conditions, the network transfer rates appear to be self-organized towards a close to optimal solution of the explore-exploit dilemma.

We are confident that the results obtained for our model suggest novel ways of analyzing and interpreting similar datasets offering elements on which to base strategic choices.

## Methods

To solve Eq. (4), one integrates the stochastic differential equations


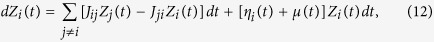


with the random noise *η*_*i*_(*t*) evolving according to


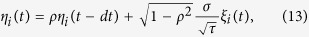


where *ρ* ≡ *e*^−*dt*/*τ*^, and *ξ*_*i*_ is Gaussian-distributed with zero mean and unit variance satisfying 〈*ξ*_*i*_(*t*) *ξ*_*j*_(*t*′)〉 = *c*_*ij*_*δ*(*t* − *t*′). Cross-correlation among the noise terms is attained by performing the LDL decomposition^26^ (a variant of Cholesky decomposition) of the matrix *C* ≡ (*c*_*ij*_)_*i*,*j*=1, …, *N*_, i.e., *C* = *LDL*^*T*^, (see, e.g.^26^) and applying it to a set of independent Gaussians 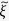 to generate 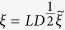. Under the natural assumption 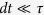 different integration schemes (e.g., Itô, Stratonovich) provide the same results.

The calibration of the four model parameters 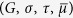 proceeds as follows. Eq. (12) can be rewritten as


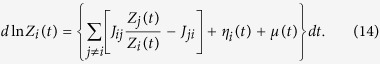


**Figure 7:**
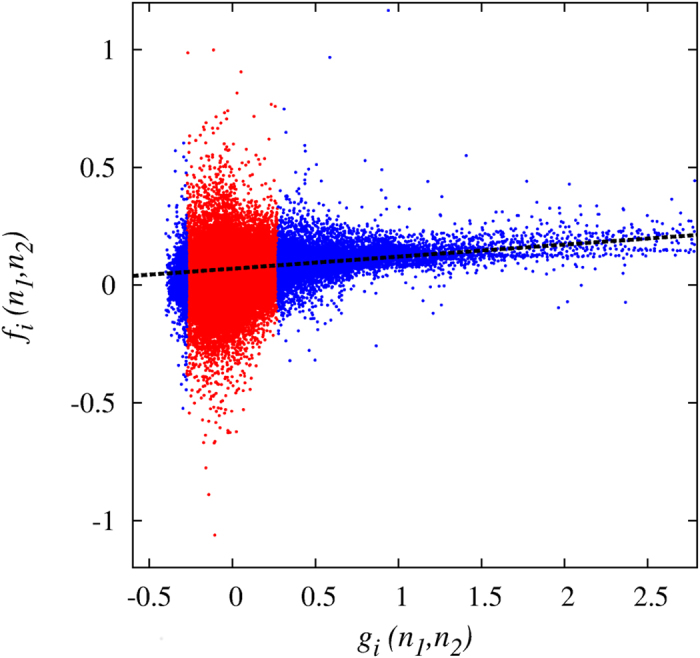
Calibration of the parameter *G*. Scatter plot of *f*_*i*_(*n*_1_, *n*_2_) vs. *g*_*i*_(*n*_1_, *n*_2_), with *i* = 1, …, 219, *n*_1_ = 0, …, 37, *n*_2_ = *n*_1_ + 1, …, 38. In blue (red) are the 10% (90%) of the points with larger (smaller) |*g*_*i*_|. The dashed line is the linear regression *y* = *Gx* + *k*, giving *G* = 0.051 and *k* = 0.069.

Integrating Eq. (14) between *n*_1_ and *n*_2_ we may write


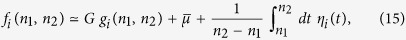


where 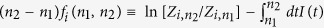 is provided by empirical data, and


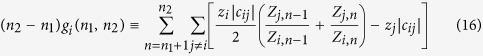


approximates through the empirical observations the stochastic integral involving the transfer terms. The last term in Eq. (15) can be viewed as a source of random errors for *f*_*i*_. As a result, the parameter *G* can be calibrated as the slope of a linear regression of *f*_*i*_ vs *g*_*i*_. However, when |*g*_*i*_| is small the random source makes the determination of *G* not reliable. Through synthetic simulations of the model with the same duration and initial conditions of the empirical data, we verified that by considering in the above regression only 1/10 of the points with larger |*g*_*i*_|, the calibration procedure obtains the correct *G*-value within a confidence of 10%. In Fig. 7 we display the scatter plot of *f*_*i*_ vs *g*_*i*_, indicating in blue the points selected for the linear regression (dashed line). In fact 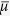 should correspond to the intercept of the regression. With our restriction to large |*g*_*i*_|, the estimate of 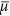 through this intercept is not very precise and becomes sharper only if all |*g*_*i*_|’s are considered. Below, we choose an alternative method for the calibration of 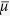 (see below).

A straightforward calculation yields


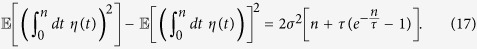


**Figure 8:**
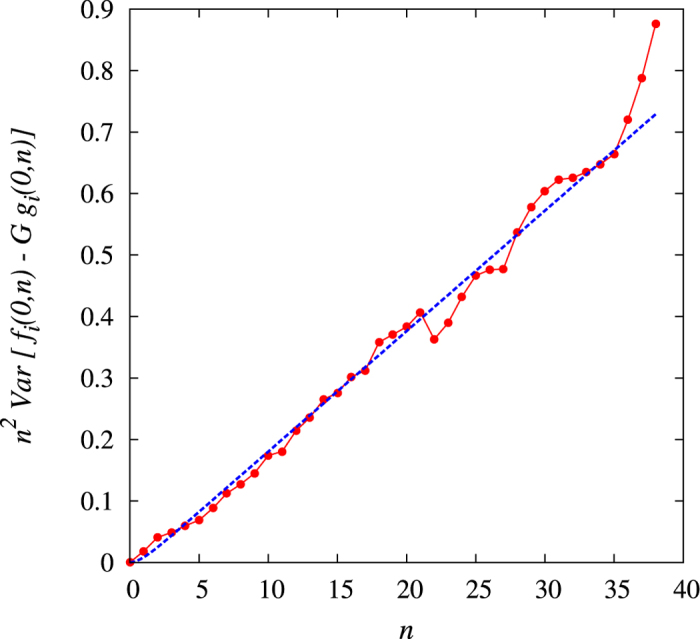
Calibration of the parameters *σ* and *τ*. Variance of *n*[ *f*_*i*_(0, *n*) − *Gg*_*i*_(0, *n*)] as a function of *n*. Dashed line is a fit with the r.h.s. of Eq. (17), giving *σ* = 0.098 and *τ* = 0.8.

We thus calibrate the parameters *σ* and *τ* by fitting with the r.h.s. of Eq. (17) the empirical variance of *n*[*f*_*i*_(0, *n*) − *Gg*_*i*_(0, *n*)], evaluated averaging over all products *i* (see Fig. 8). We remind that such a variance is independent of *μ*(*t*).

Finally, the parameter 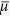 is calibrated as the best fitting value which, added to the inflation and to the growth due to the network, numerically reproduces the overall growth of real exports in the time interval [0, 38] at given calibrated values for *G, σ, τ*, starting from the historical initial conditions at *t* = 0. The values *I*(*t*) for the inflation rate are deduced from the CPI Detailed Report made by Bureau of Labor Statistics^24^ and reported in Table 1.

The application of the above procedure gives in summary


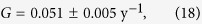



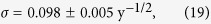



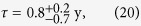



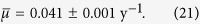


Parameter precisions are evaluated as standard deviations resulting from repeated applications of the above calibration procedure to synthetic simulations of Eq. (12), performed within the time interval [0, 38] at the calibrated value of the parameters.

## Additional Information

**How to cite this article**: Caraglio, M. *et al*. Export dynamics as an optimal growth problem in the network of global economy. *Sci. Rep.* **6**, 31461; doi: 10.1038/srep31461 (2016).

## Figures and Tables

**Table 1:** Yearly inflation rate according to^24^.

Year	*I*_*t*_	Year	*I*_*t*_	Year	*I*_*t*_	Year	*I*_*t*_
1963	1.3	1964	1.3	1965	1.6	1966	2.9
1967	3.1	1968	4.2	1969	5.5	1970	5.7
1971	4.4	1972	3.2	1973	6.2	1974	11.0
1975	9.1	1976	5.8	1077	6.5	1978	7.6
1979	11.3	1980	13.5	1981	10.3	1982	6.2
1983	3.2	1984	4.3	1985	3.6	1986	1.9
1987	3.6	1988	4.1	1989	4.8	1990	5.4
1991	4.2	1992	3.0	1993	3.0	1994	2.6
1995	2.8	1996	3.0	1997	2.3	1998	1.6
1999	2.2	2000	3.4				

The average value over the 38 years is *I* = 4.73%.
